# LncRNA RNA Component of Mitochondrial RNA-Processing Endoribonuclease Promotes AKT-Dependent Breast Cancer Growth and Migration by Trapping MicroRNA-206

**DOI:** 10.3389/fcell.2021.730538

**Published:** 2021-09-21

**Authors:** Yingdan Huang, Bangxiang Xie, Mingming Cao, Hua Lu, Xiaohua Wu, Qian Hao, Xiang Zhou

**Affiliations:** ^1^Fudan University Shanghai Cancer Center and Institutes of Biomedical Sciences, Fudan University, Shanghai, China; ^2^Department of Oncology, Shanghai Medical College, Fudan University, Shanghai, China; ^3^Beijing Institute of Hepatology, Beijing You An Hospital, Capital Medical University, Beijing, China; ^4^Beijing Engineering Research Center for Precision Medicine and Transformation of Hepatitis and Liver Cancer, Beijing, China; ^5^Department of Biochemistry & Molecular Biology, Tulane Cancer Center, Tulane University School of Medicine, New Orleans, LA, United States; ^6^Department of Gynecologic Oncology, Fudan University Shanghai Cancer Center, Fudan University, Shanghai, China; ^7^Key Laboratory of Breast Cancer in Shanghai, Fudan University Shanghai Cancer Center, Fudan University, Shanghai, China; ^8^Shanghai Key Laboratory of Medical Epigenetics, International Co-Laboratory of Medical Epigenetics and Metabolism, Ministry of Science and Technology, Institutes of Biomedical Sciences, Fudan University, Shanghai, China

**Keywords:** non-coding RNA, RMRP, miR-206, AKT, breast cancer

## Abstract

The RNA component of mitochondrial RNA-processing endoribonuclease (RMRP) was recently shown to play a role in cancer development. However, the function and mechanism of RMRP during cancer progression remain incompletely understood. Here, we report that RMRP is amplified and highly expressed in various malignant cancers, and the high level of RMRP is significantly associated with their poor prognosis, including breast cancer. Consistent with this, ectopic RMRP promotes proliferation and migration of *TP53*-mutated breast cancer cells, whereas depletion of RMRP leads to inhibition of their proliferation and migration. RNA-seq analysis reveals AKT as a downstream target of RMRP. Interestingly, RMRP indirectly elevates AKT expression by preventing AKT mRNA from miR-206-mediated targeting via a competitive sequestering mechanism. Remarkably, RMRP endorses breast cancer progression in an AKT-dependent fashion, as knockdown of AKT completely abolishes RMRP-induced cancer cell growth and migration. Altogether, our results unveil a novel role of the RMRP-miR-206-AKT axis in breast cancer development, providing a potential new target for developing an anti-breast cancer therapy.

## Introduction

The past decade has witnessed the growing importance of the non-coding RNAs (ncRNA) as critical regulators of almost all biological aspects of human cancer (Esteller, 2011; Wolin and Maquat, 2019). Long non-coding RNAs (lncRNA) and microRNAs (miRNA) constitute the majority of ncRNA (Garzon et al., 2009; Gibb et al., 2011). LncRNAs are a group of ncRNAs with >200 nucleotides (Schmitt and Chang, 2016), while miRNAs represent a group of small regulatory RNAs with 18-23 nucleotides in length (Garneau et al., 2007; Fabian et al., 2010). Three modes of action have been proposed to illustrate how lncRNAs might function in cancer (Schmitt and Chang, 2016; He et al., 2019). First, the nuclear lncRNAs can modulate gene expression by controlling local chromatin remodeling or directing the recruitment of regulatory factors to specific promoter regions on chromosomes. One prominent example is the p53-inducible large intergenic non-coding RNA (lincRNA)-p21 that recruits hnRNP-K to the proper genomic locations to globally repress gene transcription (Huarte et al., 2010). Also, lncRNAs can interact with multiple proteins to facilitate the formation of functional complexes or perturb molecule interactions. For instance, lncRNA-ROR was shown to inhibit p53 translation by binding to hnRNP-I and preventing the interaction of the latter with p53 mRNA (Zhang et al., 2013). Moreover, lncRNAs can associate with different RNA molecules, such as mRNAs and microRNAs, to regulate mRNA turnover and translation. It has been shown that a number of highly expressed lncRNAs are able to act as competitive endogenous RNAs (ceRNAs) to sequester microRNAs (miRNAs) away from their mRNA targets (Tay et al., 2014). Because of the increasingly complex network with the addition of ncRNAs in cancer, more efforts are needed to thoroughly dissect the molecular basis underlying the role of lncRNAs in disease development.

The PI3K/AKT signaling pathway plays an important role in cell fate decisions, including growth and proliferation, survival, angiogenesis, metabolic remodeling, and chemoresistance (Hoxhaj and Manning, 2020; Liu et al., 2020). Recently, lncRNAs have been shown to play a critical role in the AKT pathway (Peng et al., 2017; Revathidevi and Munirajan, 2019). LncRNA AK023948 was found to functionally interact with DHX9 and the regulatory subunit of PI3K, p85, leading to AKT activation (Koirala et al., 2017). LINK-A could facilitate AKT and Phosphatidylinositol 3,4,5-trisphosphate [PtdIns(3,4,5)P_3_] interaction and, as thus, induce enzymatic activation of AKT by forming a trimeric complex (Lin et al., 2017). PCAT1 interacted directly with FKBP51, thus perturbing the PHLPP/FKBP51 complex that is required for dephosphorylation of AKT at Ser-473 (Shang et al., 2019). In this study, we identified a lncRNA, the RNA component of mitochondrial RNA-processing endoribonuclease (RMRP), as an additional regulator of the AKT pathway as described below.

RMRP was found to be involved in the cleavage of the RNA primer for mitochondrial DNA replication (Chang and Clayton, 1987) and the precursor of ribosomal RNA (rRNA) (Goldfarb and Cech, 2017). Mutation of RMRP was identified in patients with cartilage-hair hypoplasia, a human ribosomopathy characterized by metaphyseal dysplasia, anemia, and immune dysregulation (Ridanpaa et al., 2001; Narla and Ebert, 2010). RMRP also plays a role in cancer development. Mutations in the *RMRP* promoter led to enhanced nuclear protein binding to the promoter, consequently elevating transcription of *RMRP*, which might be associated with cancer progression (Rheinbay et al., 2017; Son et al., 2019). Moreover, RMRP has been shown to act as a sponge for microRNAs and promote gastric and lung cancer development (Meng et al., 2016; Shao et al., 2016; Hussen et al., 2021). We recently found that RMRP interacts with and sequesters SNRPA1 in the nucleus, where the latter binds to wild type p53 (wt p53) and promotes MDM2-mediated proteasomal degradation of wt p53 in colorectal cancer (Chen et al., 2021). In breast cancer, upregulation of RMRP partially resulted from its promoter mutation (Rheinbay et al., 2017) or Wnt/Hippo activation (Park and Jeong, 2015), but its biological function and the underlying mechanism in this cancer remain unclear. Herein, we report the wt p53-independent tumor-promoting function of RMRP. We found that the RMRP gene is amplified and overexpressed in a variety of human cancers, and the high level of RMRP is significantly associated with poor prognosis of multiple cancers, including breast cancer. Remarkably, RMRP promoted proliferation and migration of *TP53*-mutated breast cancer cells by activating the AKT signaling pathway. It did so by preventing miRNA-206 from binding to its target AKT mRNA. Our study establishes a role of the RMRP-miR-206-AKT axis in breast cancer development, and provides these molecules as potential biomarkers and therapeutic targets for future developing treatments of the disease.

## Materials and Methods

### Cell Culture and Transient Transfection

Human breast cancer cell lines JIMT-1 and BT549 were cultured in Dulbecco’s modified Eagle’s medium supplemented with 10% fetal bovine serum, 100 U/ml penicillin, and 100 μg/ml streptomycin. All cells were cultured at 37°C in an incubator containing 5% carbon dioxide. Cells were seeded on the dish at appropriate density one day before transfection, and then transfected plasmids, siRNAs or the miRNA mimic/inhibitor according to the manufacturer’s protocol of the Hieff Trans liposomal transfection reagent (Yeasen, shanghai, China). Cells were harvested at 24–48 h post transfection for immunoblotting or RT-quantitative PCR (qPCR) analyses.

### Plasmids, siRNAs, and miRNA Mimics and Inhibitor

The plasmid expressing RMRP was purchased from Shanghai Genechem (Shanghai, China). The pcDNA3-luc-mcs Dual-Luciferase miRNA Target Expression Vector was a gift from Shenglin Huang. pcDNA3-luc-mcs-AKT-3′UTR reporter plasmids were generated by inserting the AKT 3′UTR amplified by PCR into the pcDNA3-luc-mcs vector. SiRNAs targeting RMRP and AKT were designed by the BLOCK-iT^TM^ RNAi Designer^1^ and synthesized by Genepharma (Shanghai, China). The siRNA sequences are as follows, siNC: UUCUCCGAACGUGUCACGU, siRMRP-1: CCUAG GCUACACACUGAGGACU, siRMRP-2: GUUCGUGCUGAA GGCCUGUAU, siAKT-1: GCACCUUCAUUGGCUACAA, siAKT-2: GCGUGACCAUGAACGAGUU. The miRNA mimics and inhibitors were designed and synthesized by Genepharma. The sequences are as follows, double-stranded hsa-miR-206 mimics: UGGAAUGUAAGGAAGUGUGUGG and ACACACUUCCUUACAUUCCAUU; and hsa-miR-206 inhibitor: CCACACACUUCCUUACAUUCCA.

### Generation of Lentiviral Particles

The PWPXL-RMRP plasmid was generated by inserting the full-length sequence of RMRP into the lentivirus-based PWPXL vector. HEK293T cells were transfected with PWPXL-vector or PWPXL-RMRP, along with the packaging plasmid psPAX2 and the envelope plasmid pMD2.G. The virus particles were collected 48 h after transfection. The supernatant of 2-4 ml containing virus particles was added to breast cancer cells, JIMT-1 and BT549, for 24-36 h.

### CRISPR/Cas9-Mediated Gene Editing

The CRISPR/Cas9 targeting vector lentiCRISPR v2 was purchased from Addgene (Cambridge, MA, United States). The sgRNA for RMRP was designed at http://crispr.mit.edu/, and was cloned into the lentiCRISPR v2 vector at the BsmBI site. The combination of sgRNAs was used to achieve the best efficiency as previously described (Chen et al., 2021), and two different clones, RMRP-KO#1 and RMRP-KO#2, were selected for future experiments. The cells were infected with the lentiviruses encoding the sgRNAs and selected by 1 μg/ml puromycin for a week.

### Reverse Transcription and Quantitative PCR Analysis

Total RNAs were isolated from cells using RNAiso Plus (Takara, Dalian, China) following the manufacturer’s protocol. Total RNAs of 0.5 to 1 μg were used as templates for reverse transcription using the PrimeScript^TM^ RT reagent Kit with gDNA Eraser (Takara, Dalian, China). Quantitative RT-PCR (RT-qPCR) was conducted using TB Green^TM^ Premix according to the manufacturer’s protocol (Takara, Dalian, China). The RT-qPCR primers GAPDH, U6, RMRP and AKT are as follows, GAPDH: 5′-GGAGCGAGATCCCTCCAAAAT-3′ and 5-′GGCTGTTGTCATACTTCTCATGG-3′, U6: 5′-GCTTCGG CAGCACATATACTAAAAT-3′ and 5′-CGCTTCACGAATTTG CGTGTCAT-3′, RMRP: 5′-TGCTGAAGGCCTGTATCCT-3′ and 5′-TGAGAATGAGCCCCGTGT-3′, and AKT: 5′- AGCGA CGTGGCTATTGTGAAG -3′ and 5′- GCCATCATTCTTGAG GAGGAAGT-3′.

### Immunoblotting

Cells were harvested and lysed in lysis buffer consisting of 50 mM Tris/HCl (pH7.5), 0.5% Nonidet P-40 (NP-40), 1 mM EDTA, 150 mM NaCl, 1 mM dithiothreitol (DTT), 0.2 mM phenylmethylsulfonyl fluoride (PMSF), 10 μM pepstatin A and 1 μg/ml leupeptin. Equal amounts of clear cell lysate (20–80 μg) were used for immunoblotting (IB) analysis as described previously (Zhou et al., 2013). anti-GAPDH (Catalog No. 60004-1, Proteintech), anti-AKT (Catalog No. #9272, Cell Signaling Technology, Danvers, MA, United States), anti-pAKT (Thr-308) (Catalog No. #4056, Cell Signaling Technology, Danvers, MA, United States), and the secondary antibodies for rabbit (Catalog No. ARG65351, Arigo) and mouse (Catalog No. ARG65350, Arigo) were commercially purchased.

### Cell Viability Assay

To detect cell proliferation, the Cell Counting Kit-8 (CCK-8) (Dojindo Molecular Technologies, Japan) was used according to the manufacturer’s instructions. Cells (2000–5000) were seeded per well in 96-well culture plates at 12 h post transfection. Cell viability was determined by adding WST-8 at a final concentration of 10% to each well, and the absorbance of the samples was measured at 450 nm using a Microplate Reader every 24 h for 4–5 days.

### Transwell Invasion Assay

The assay was performed using the transwell chamber inserts in a 24-well plate. Briefly, 5 × 10^4^ cells suspended in 200 μl of serum-free medium were added to the upper chamber after 12 h post transfection. The lower chambers were filled with the culture medium with 20% fetal bovine serum. After culture for 24–36 h at 37°C, the cells on the upper surface were scraped and washed away, while the cells on the lower surface were fixed with methanol and stained with 0.1% crystal violet. The number of invasive cells was counted in at least three randomly selected fields under an optical microscope by image J software.

### Luciferase Reporter Assay

HEK293T cells were seeded at 5 × 10^3^ cells per well in 96 well plates. The cells were then co-transfected with the combinations of the Renilla plasmid, the pcDNA3-luc-mcs-AKT-3′UTR reporter plasmid, PWPXL or PWPXL-RMRP plasmid, and the control or miR-206 mimics as indicated in the figure. At 48 h post transfection, cells were lysed using passive lysis buffer, and the Firefly and Renilla luciferase activities were measured by the dual-luciferase assay kit (Promega, Madison, WI, United States).

### Databases of Cancer Patients

The ciBioPortal website was used for analyzing the mutation and copy number variations of RMRP based on the TCGA database (Cerami et al., 2012; Gao et al., 2013). The raw data of gene expression were available in Gene Expression Omnibus database (GSE76250) (Jiang et al., 2016). Cancer patient survival was analyzed by the Kaplan-Meier Plotter website (Nagy et al., 2021).

### Statistics

Statistical analyses were performed using GraphPad Prism 6 software or SPSS 19.0 software. Data of experiments are expressed as mean ± standard deviation (SD) of at least three independent experiments. The Student’s *t* test or one-way analysis of variance was performed to evaluate the differences between two groups or more than two groups. The Kaplan–Meier statistics were used to analyze the significant difference of patient survival. *p* < 0.05 was considered statistically significant, and the asterisks represent significance in the following way: ^∗^*p* < 0.05, ^∗∗^*p* < 0.01, and **^∗∗∗^***p* < 0.001.

## Results

### RNA Component of Mitochondrial RNA-Processing Endoribonuclease Is Associated With Unfavorable Prognosis in Different Cancers

**FIGURE 1:**
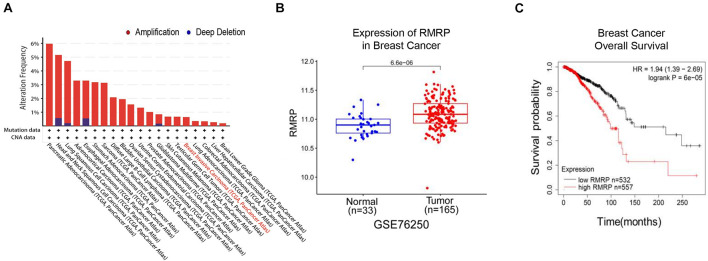
RMRP is associated with poor survival of breast cancer patients. **(A)** The genomic alterations of the *RMRP* gene in various human cancers from the TCGA database. **(B)** RMRP is expressed at a higher level in breast cancer compared to normal tissues from the GEO database. **(C)** The Kaplan-Meier analysis shows that high expression of RMRP is correlated with unfavorable overall survival of breast cancer.

To explore the clinical relevance of RMRP in human cancers, we analyzed the TCGA database and found that the *RMRP* gene is amplified in multiple cancers (Figure 1A). Consistently, the expression of RMRP was preferentially upregulated in cancerous tissues compared to normal tissues (Figure 1B and Supplementary Figure 1A) by mining the Gene Expression Omnibus (GEO) and UALCAN databases (Chandrashekar et al., 2017). In addition, the Kaplan–Meier analysis revealed that the increased expression of RMRP is significantly associated with unfavorable prognosis of various human cancers, including breast cancer, head-neck carcinoma, lung adenocarcinoma, pancreatic ductal adenocarcinoma, stomach adenocarcinoma, and uterine corpus endometrial carcinoma (Figure 1C and Supplementary Figure 1B). Therefore, these findings suggest that RMRP may play an oncogenic role in cancer.

### RNA Component of Mitochondrial RNA-Processing Endoribonuclease Promotes Proliferation and Migration of Breast Cancer Cells

**FIGURE 2:**
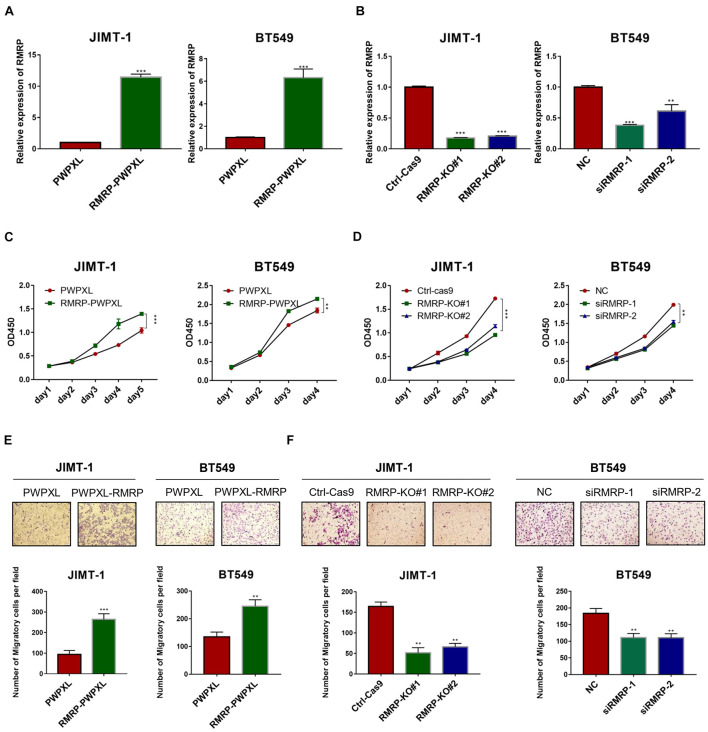
RMRP promotes proliferation and migration of breast cancer cells. **(A)** Efficiency of RMRP-PWPXL overexpression in JIMT-1 and BT549 cells was evaluated by RT-qPCR. **(B)** Efficiency of CRISPR-Cas9-mediated knockout or RNAi-mediated knockdown of RMRP in JIMT-1 and BT549 cells was evaluated by RT-qPCR. **(C)** Overexpression of RMRP prompts JIMT-1 and BT549 cell proliferation determined by the CCK-8 assay. **(D)** Depletion of RMRP represses JIMT-1 and BT549 cell proliferation determined by the CCK-8 assay. **(E)** Overexpression of RMRP prompts JIMT-1 and BT549 cell migration determined by the Transwell assay. **(F)** Depletion of RMRP prompts JIMT-1 and BT549 cell migration determined by the Transwell assay. **p < 0.01, ***p < 0.001.

Since we previously showed that RMRP endorses cancer cell growth by inhibiting the wt p53 pathway (Chen et al., 2021), we wanted to determine if RMRP can function independently of wt p53 or not. To do so, we selected the *TP53*-mutated breast cancer cell lines, JIMT-1 and BT549, as the model systems here by generating RMRP-stably overexpressing cell lines (Figure 2A). Interestingly, ectopic RMRP still significantly promoted the survival of these breast cancer cells (Figure 2C). Conversely, using RMRP-knockout or -knockdown JIMT-1 and BT549 cells (Figure 2B), we found that depletion of RMRP significantly suppresses the growth of these cells (Figure 2D). Of note, knockout of RMRP via CRISPR-Cas9 achieved a more profound inhibitory effect on cancer cell growth (Figure 2D), because RMRP expression was more markedly depleted in JIMT-1 cells (Figure 2B). Furthermore, we found that ectopic expression of RMRP dramatically promotes, while depletion of RMRP prohibits, breast cancer cell migration (Figures 2E,F). Finally, we examined RMRP’s function in wt p53-harboring breast cancer cells. As shown in Supplementary Figure 2, overexpression of RMRP significantly boosted, while knockdown of RMRP repressed, growth and migration of MCF-7 (Supplementary Figures 2A,B) and CAL-51 cells (Supplementary Figures 2C,D). Together, these results demonstrate that RMRP can promote proliferation and migration of breast cancer cells independently of wt p53.

### RNA Component of Mitochondrial RNA-Processing Endoribonuclease Activates the AKT Pathway by Upregulating Its Expression

**FIGURE 3:**
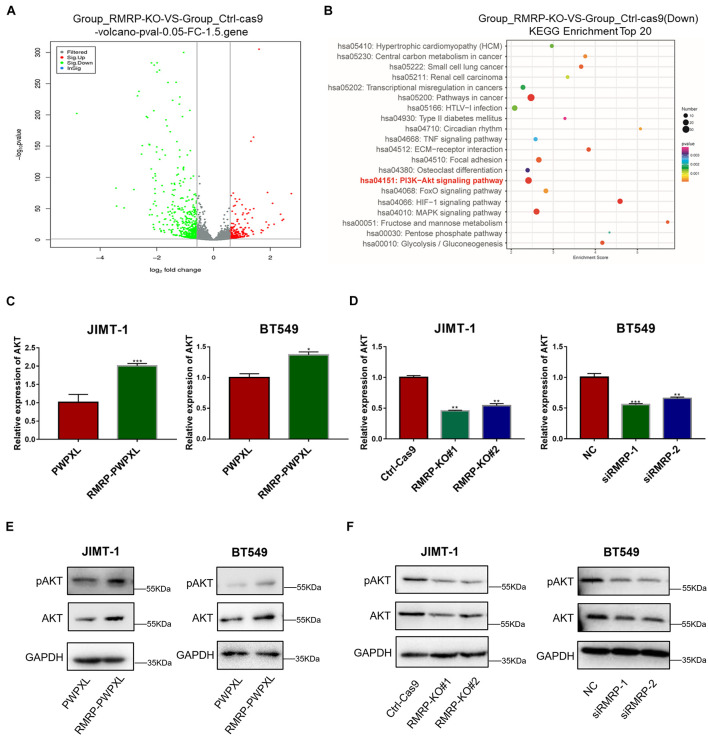
RMRP upregulates AKT expression. **(A)** Differentially expressed gene profile in response to RMRP knockout revealed by the volcano-map. **(B)** Downregulated cancer-related pathways in response to RMRP knockout revealed by KEGG enrichment. **(C)** Overexpression of RMRP elevates AKT mRNA expression in JIMT-1 and BT549 cells. **(D)** Depletion of RMRP reduces AKT mRNA expression in JIMT-1 and BT549 cells. **(E)** Overexpression of RMRP elevates AKT protein expression and phosphorylated AKT in JIMT-1 and BT549 cells. **(F)** Depletion of RMRP reduces AKT protein expression and phosphorylated AKT in JIMT-1 and BT549 cells. *p < 0.05, **p < 0.01, ***p < 0.001.

To explore the molecular mechanism underlying the wt p53-independent oncogenic effects of RMRP, we re-analyzed the RNA-seq results from HCT116 cells as reported in our previous study (Chen et al., 2021) and found that a myriad of genes are dysregulated in response to RMRP knockout (Figure 3A). The Kyoto Encyclopedia of Genes and Genomes (KEGG) analysis revealed the AKT signaling pathway as the most significantly downregulated in RMRP-depleted cells (Figure 3B). To validate this result, we determined if RMRP regulates the mRNA expression of AKT by RT-qPCR in breast cancer cells. Indeed, as shown in Figure 3C, overexpression of RMRP increased the level of AKT mRNA. Consistently, depletion of RMRP reduced the AKT mRNA level (Figure 3D). Consistently, ectopic RMRP dramatically increased the level of AKT protein as well as its phosphorylated form in JIMT-1 and BT549 cells (Figure 3E), while knockout or knockdown of RMRP resulted in the significant reduction of AKT and phosphorylated AKT in both breast cancer cell lines (Figure 3F). Collectively, these results demonstrate that RMRP can activate the AKT signaling pathway, which might account for its tumor-promoting functions in breast cancer cells.

### RNA Component of Mitochondrial RNA-Processing Endoribonuclease Induces AKT Expression by Sequestering miR-206

**FIGURE 4:**
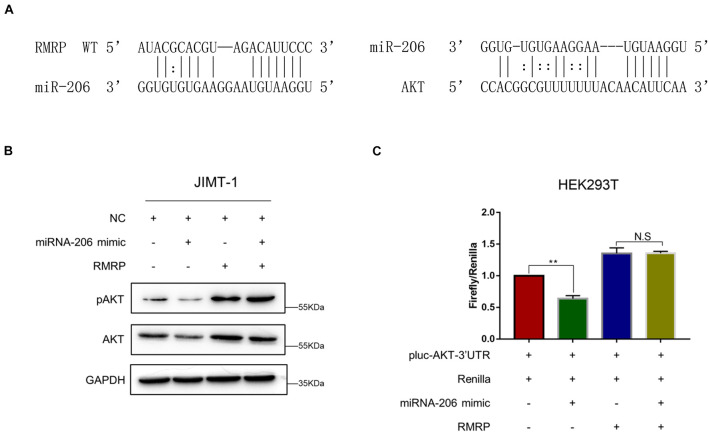
RMRP upregulates AKT expression by sponging miR-206. **(A)** Both RMRP and AKT are potentially targeted by miR-206 through the miRTarBase. **(B)** miR-206 mimics-induced inhibition of AKT can be completely restored by RMRP overexpression. **(C)** The luciferase reporter assay was performed to show that RMRP overexpression completely abrogates miR-206 mimics-induced inhibition of the reporter gene fused with the AKT 3′-UTR. **p < 0.01.

Next, we wanted to determine how RMRP promotes AKT expression. Since miRNAs have been documented to target mRNAs for degradation and/or inhibit their translation (Bartel, 2009), and also, lncRNAs can derepress mRNA expression by sequestering these inhibitory miRNAs (Tay et al., 2014), we decided to test if RMRP might also utilize this mechanism to activate the expression of AKT. By searching the microRNA-target interaction database, miRTarBase (Huang et al., 2020), we identified miR-206 as a potential binder for both RMRP and AKT (Figure 4A). To test this, we ectopically expressed miR-206 mimics in JIMT-1 cells, and found that the levels of AKT and phosphorylated AKT are indeed reduced (Figure 4B). Remarkably, overexpression of RMRP completely abrogated miR-206-mediated AKT inhibition (Figure 4B). Moreover, miR-206 mimics significantly repressed the expression of the luciferase reporter gene harboring the AKT 3′-UTR (Figure 4C). Consistently, overexpression of RMRP abolished miR-206 inhibition of the luciferase activity (Figure 4C). Taken together, these results demonstrate that RMRP induces AKT protein levels by overcoming miR-206’s inhibition of its expression.

### RNA Component of Mitochondrial RNA-Processing Endoribonuclease Negates miR-206-Mediated Repression of Breast Cancer Cell Growth and Migration

**FIGURE 5:**
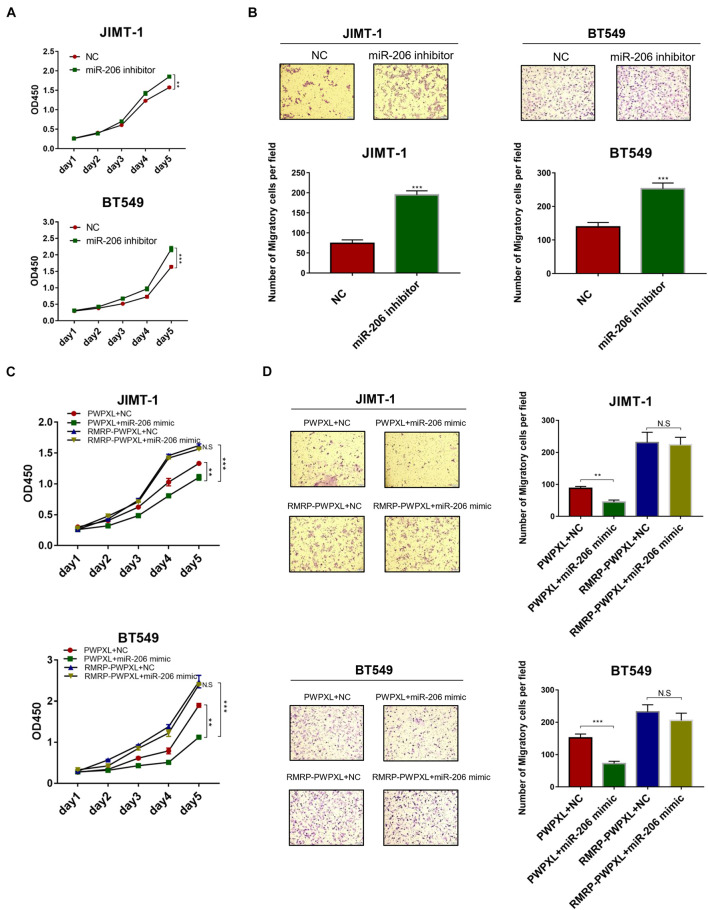
RMRP negates the tumor suppressive function of miR-206. **(A)** Inhibition of miR-206 promotes JIMT-1 and BT549 cell proliferation determined by the CCK-8 assay. **(B)** Inhibition of miR-206 promotes JIMT-1 and BT549 cell migration determined by the Transwell assay. **(C)** RMRP overexpression abrogates miR-206 inhibition of cell proliferation determined by the CCK-8 assay. **(D)** RMRP overexpression abrogates miR-206 inhibition of cell migration determined by the Transwell assay. **p < 0.01, ***p < 0.001.

Given that miR-206 inhibits AKT expression (Figures 4B,C), we next tested if miR-206 suppresses breast cancer cell growth and migration. Delivery of the miR-206 inhibitor into JIMT-1 and BT549 cells significantly triggered their proliferation (Figure 5A). Consistently, the miR-206 inhibitor promoted JIMT-1 and BT549 cell migration (Figure 5B). Also, miR-206 mimics significantly reduced JIMT-1 and BT549 cell proliferation and migration (Figures 5C,D), indicating that miR-206 plays a tumor suppressive role in breast cancer. Importantly, RMRP overexpression completely abolished miR-206’s tumor suppressive activity (Figures 5C,D). These results demonstrate that RMRP endorses breast cancer development by counteracting the tumor suppression function of miR-206.

### RNA Component of Mitochondrial RNA-Processing Endoribonuclease Promotes Breast Cancer Cell Survival and Migration in an AKT-Dependent Fashion

**FIGURE 6:**
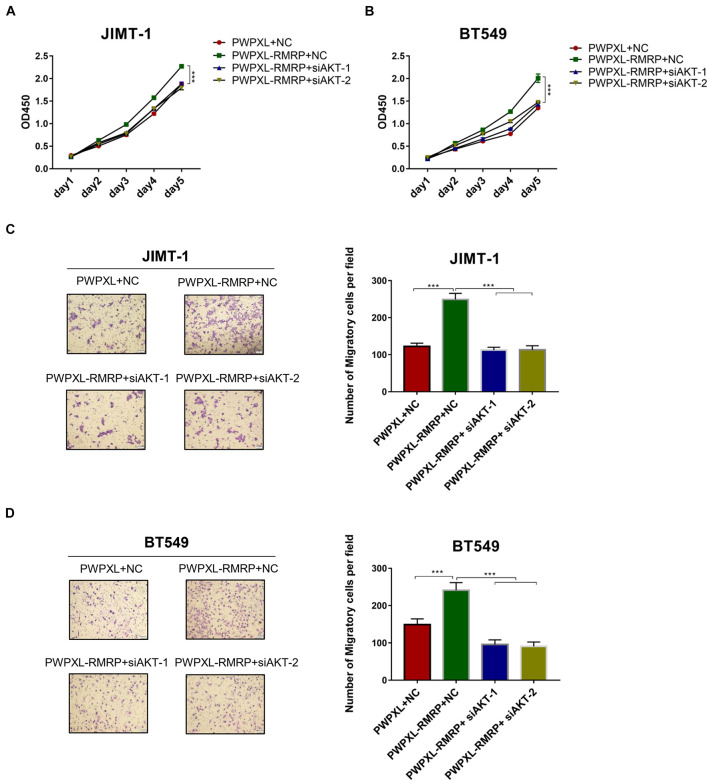
Ectopic RMRP promotes breast cancer cell growth and migration dependently of AKT. **(A)** Knockdown of AKT restores RMRP-induced JIMT-1 cell proliferation determined by the CCK-8 assay. **(B)** Knockdown of AKT restores RMRP-induced BT549 cell proliferation determined by the CCK-8 assay. **(C)** Knockdown of AKT abolishes RMRP-induced JIMT-1 cell migration determined by the Transwell assay. **(D)** Knockdown of AKT abolishes RMRP-induced BT549 cell migration determined by the Transwell assay. ***p < 0.001.

Finally, we determined if RMRP promotes breast cancer cell growth and migration through activation of the AKT pathway. The cell viability assay revealed that ectopic RMRP significantly increases cancer cell growth, while knockdown of AKT by two independent siRNAs blocks the tumor-promoting function of RMRP in JIMT-1 and BT549 cells (Figures 6A,B). In addition, the migratory potential of cancer cells was also evaluated through the transwell assay. Consistently, ectopic RMRP dramatically enhanced spread of JIMT-1 and BT549 cells, whereas depletion of AKT completely abrogated RMRP-induced cancer cell migration (Figures 6C,D). Taken together, these results demonstrate that activation of the AKT pathway is required for RMRP-mediated breast cancer survival and migration independently of wt p53.

## Discussion

**FIGURE 7:**
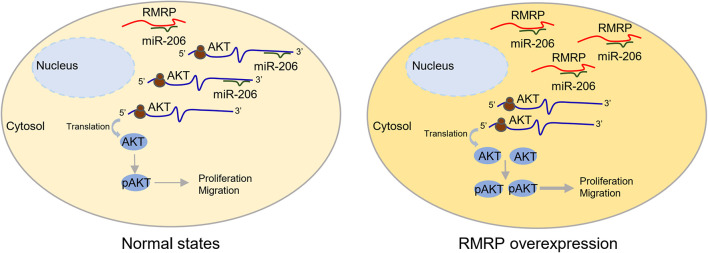
A schematic for RMRP induces AKT expression by inhibiting miR-206 in breast cancer. Under the physiological condition, miR-206 targets AKT mRNA for rapid turnover or translational inhibition, leading to reduced cell proliferation and migration (left panel). In the context of RMRP overexpression, RMRP sequesters miR-206 to unleash AKT activity, leading to enhanced breast cancer cell proliferation and migration (right panel).

RMRP has been shown to promote progression of various cancers, including colorectal cancer (Chen et al., 2021), gastric cancer (Shao et al., 2016), lung cancer (Meng et al., 2016), and cholangiocarcinoma (Tang et al., 2019). Recently, it was reported that recurrent mutations in the *RMRP* promoter are associated with higher expression level of RMRP in breast cancer, suggesting that this lncRNA may play a role in breast carcinogenesis (Rheinbay et al., 2017). In this study, we found that RMRP is amplified and overexpressed in numerous human cancers including breast cancer, and its high expression level is significantly associated with unfavorable cancer prognosis (Figure 1 and Supplementary Figure 1). We then verified RMRP as an oncogenic lncRNA, because it could drive breast cancer cell growth and migration (Figure 2 and Supplementary Figure 2). Mechanistically, RMRP can activate the AKT signaling pathway as demonstrated by RNA-seq, RT-qPCR and immunoblotting analyses (Figure 3). Remarkably, RMRP induces AKT level and activity by preventing miR-206-mediated inhibition of AKT mRNA expression (Figure 4). Hence, our study as presented above unveils a critical role of the RMRP-miR-206-AKT cascade in breast cancer cell growth and migration (Figures 5–7).

Activation of the PI3K/AKT pathway is crucial to tumor growth and propagation. In response to insulin, growth factors or cytokines, the lipid kinase PI3K can be recruited to the plasma membrane. PI3K then phosphorylates phosphatidylinositol 4,5-bisphosphate [PtdIns(4,5)P_2_] to produce PtdIns(3,4,5)P_3_ that serves as a second messenger to recruit AKT to the membrane, where it is fully activated through phosphorylation at Thr-308 and Ser-473 (Alessi et al., 1996, 1997). In general, activated AKT promotes cancer development via phosphorylation and inhibition of the three key downstream effectors, GSK3 (Cross et al., 1995), TSC2 (Menon et al., 2014), and FOXO (Brunet et al., 1999). The first identified AKT substrate, GSK3, was found to mediate phosphorylation of c-MYC, SREBP, NRF2, and HIF1α, leading to proteasomal degradation of these oncoproteins. GSK3 is inactivated through phosphorylation by AKT, which leads to derepression of these oncogenic transcription factors and consequent cancer growth and progression (Hoxhaj and Manning, 2020). Phosphorylation of TSC2 by AKT activated the mTORC1-S6K/4E-BP pathway that is regarded as an energy-sensor to monitor metabolic changes and support cancer cell growth (Valvezan and Manning, 2019), while phosphorylation of FOXO transcription factors enhanced tumorigenesis by influencing glycolysis, redox homeostasis, and many other cell growth-associated pathways (Hornsveld et al., 2018). Thus, our finding has emphasized the important role of RMRP by activating AKT in breast cancer.

RMRP was originally identified as a causative gene for cartilage-hair hypoplasia, because numerous mutations in the *RMRP* gene caused the disease by affecting multiple organ systems (Ridanpaa et al., 2001, 2002; Bonafe et al., 2002). Later studies revealed that mutation or loss of expression of RMRP results in the impairment of ribosome biogenesis and deregulation of the cyclin-dependent cell cycle progression, eventually leading to growth inhibition of the chondrocytic and lymphocytic cell lineages (Hermanns et al., 2005; Thiel et al., 2005). Indeed, genetic mouse models further verified the essential role of RMRP during early embryonic development, as homozygous inactivation of RMRP caused embryonic lethality (Rosenbluh et al., 2011). Additionally, it was found that human telomerase reverse transcriptase (hTERT) associates with RMRP to form a distinct ribonucleoprotein complex that has RNA-dependent RNA polymerase (RdRP) activity (Maida et al., 2009). RMRP could be thus processed into a double-stranded RNA with a hairpin structure and consequently endogenous siRNAs that are important for proper development and differentiation of skeletal, hair, and hematopoietic cells (Maida et al., 2013; Rogler et al., 2014). Recently, RMRP was also found to be involved in cancer development. The oncogenic transcription factors, such as β-catenin, YAP, and c-MYC, can activate the transcription of RMRP (Park and Jeong, 2015; Xiao et al., 2019) that promotes tumor cell survival and propagation by regulating the expression of, for example, Cyclin D2 and KRAS (Meng et al., 2016; Shao et al., 2016). Importantly, the existence of RMRP in the blood plasma exhibits a diagnostic value for detection of lung cancer (Leng et al., 2018; Yuan et al., 2020). However, the role of RMRP in breast cancer remains unclear. We previously found that RMRP can moderately promote proliferation of p53-deficient HCT116 cells, suggesting a p53-independent role of RMRP, but the underlying mechanism was elusive. In this study, we demonstrate that this role is executed by sequestering miR-206 from targeting AKT mRNA for degradation, consequently leading to AKT-dependent breast cancer cell growth and migration. Similarly, RMRP can act as a sponge of miR-206 in other types of cancer (Meng et al., 2016; Shao et al., 2016). Although recent studies suggested a potential role of RMRP in regulating AKT in the ischemic models (Kong et al., 2019; Li and Sui, 2020), our study demonstrates for the first time that RMRP can promote breast cancer cell growth and migration via AKT activation.

miR-206 is a vertebrate-specific microRNA that is involved in a variety of human diseases, including skeletal and muscular developmental disorders, heart failure, chronic obstructive pulmonary disease, Alzheimer’s disease, and numerous types of cancer (Novak et al., 2014). Studies showed that miR-206 can target estrogen receptor α (ERα), leading to inhibition of the estrogen signaling pathway and as thus cell growth and proliferation in breast cancer (Adams et al., 2007, 2009). miR-206 also suppresses stem-like and metastatic features of breast cancer by regulating the TWF1- MKL1-IL11 pathway (Samaeekia et al., 2017). In line with these findings, we also showed that miR-206 mimics repress JIMT-1 and BT549 cell growth and migration (Figures 5C,D), while the miR-206 inhibitor drives proliferation and migration of these cells (Figures 5A,B). Although a few studies suggested that miR-206 indirectly regulates the PI3K/AKT pathway by targeting c-Met or HDAC6 (Liu et al., 2017; Tang et al., 2017; Dai et al., 2018), our study identified AKT as a direct target gene of miR-206, because miR-206 mimics via its seed region significantly reduced the expression of AKT and the luciferase reporter gene fused with the 3′-UTR of AKT (Figures 4B,C). Therefore, these results reveal an uncharacterized tumor suppressive role of miR-206 by targeting the AKT pathway.

Our results indicate that the oncogenic effect of RMRP on JIMT-1 and BT549 cells largely relies on AKT activation. Given the fact that the expression of miR-206 can be repressed by ERα (Adams et al., 2007), both JIMT-1 and BT549 that were derived from ER- or triple-negative breast cancer patients (Tanner et al., 2004; Grigoriadis et al., 2012; Tian and Zhang, 2018) should have high expression level of miR-206. Also, because the expression of ER is extremely low in both breast cancer cell lines, miR-206 may preferentially target AKT for rapid turnover, as evidenced by the immunoblotting analysis above (Figure 4B). In this scenario, miR-206 thereby plays a critical role in restricting the AKT activity. Remarkably, our data clearly demonstrate that RMRP can overcome miR-206 inhibition of AKT (Figures 4B,C, 5C,D) and, as thus, trigger AKT-dependent growth and migration of breast cancer cells (Figure 6).

## Conclusion

RMRP was recently found to play a role in cancer development, but its function and the underlying mechanism in breast cancer are largely unknown. Our study uncovers the RMRP-miR-206-AKT regulatory axis as a new pathway that plays a critical role in promoting the growth and migration of aggressive breast cancer cells, which could serve as a potential target pathway for future development of prognostic biomarkers or therapeutic strategies for this type of cancer.

## Data Availability Statement

The original contributions presented in the study are included in the article/Supplementary Material, further inquiries can be directed to the corresponding authors.

## Ethics Statement

The study was approved by the Ethics Committee of Fudan University Shanghai Cancer Center.

## Author Contributions

YH conducted and analyzed most of the experiments. BX conducted and analyzed part of the experiments and provided critical reagents and materials. MC performed part of IB analysis. HL and XW provided important instructions. QH and XZ conceived, designed and supervised the study, and analyzed the data. XZ drafted and edited the manuscript. HL edited the manuscript. All authors contributed to the article and approved the submitted version.

## Conflict of Interest

The authors declare that the research was conducted in the absence of any commercial or financial relationships that could be construed as a potential conflict of interest.

## Publisher’s Note

All claims expressed in this article are solely those of the authors and do not necessarily represent those of their affiliated organizations, or those of the publisher, the editors and the reviewers. Any product that may be evaluated in this article, or claim that may be made by its manufacturer, is not guaranteed or endorsed by the publisher.

## Footnotes

1
http://rnaidesigner.thermofisher.com/rnaiexpress/design.do
