# Supplementary material for: LncRNA RNA Component of Mitochondrial RNA-Processing Endoribonuclease Promotes AKT-Dependent Breast Cancer Growth and Migration by Trapping MicroRNA-206
Source: Front Cell Dev Biol. 2021 Sep 21;9:730538. doi: 10.3389/fcell.2021.730538 (PMC8490808; doi:10.3389/fcell.2021.730538)
Supplement: Supplementary file 1 [file Data_Sheet_1.PDF]

LncRNA RMRP promotes AKT-dependent breast cancer growth and migration by trapping microRNA-206

Yingdan Huang [1,2,#], Bangxiang Xie [3,4,#], Mingming Cao [1,2], Hua Lu [5], Xiaohua Wu [2,6], Qian Hao [1,2,*], and Xiang Zhou [1,2,7,8,*]

[1] Fudan University Shanghai Cancer Center and Institutes of Biomedical Sciences, Fudan University, Shanghai 200032, China

[2] Department of Oncology, Shanghai Medical College, Fudan University, Shanghai 200032, China

[3] Beijing YouAn Hospital, Capital Medical University, Beijing Institute of Hepatology, Beijing 100069, China

[4] Beijing Precision Medicine and Transformation Engineering Technology Research Center of Hepatitis and Liver Cancer, Beijing 100069, China

[5] Department of Biochemistry & Molecular Biology and Tulane Cancer Center, Tulane University School of Medicine, New Orleans, LA 70112, USA

[6] Department of Gynecologic Oncology, Fudan University Shanghai Cancer Center, Fudan University, Shanghai 200032, China

[7] Key Laboratory of Breast Cancer in Shanghai, Fudan University Shanghai Cancer Center, Fudan University, Shanghai, 200032, China

[8] Shanghai Key Laboratory of Medical Epigenetics, International Co-laboratory of Medical Epigenetics and Metabolism, Ministry of Science and Technology, Institutes of Biomedical Sciences, Fudan University, Shanghai 200032, China

[#] Equal contribution

[*] Correspondence:

Qian Hao, Fudan University Shanghai Cancer Center, Fudan University, Shanghai 200032, China. Email: haoqian@fudan.edu.cn

Xiang Zhou, Fudan University Shanghai Cancer Center and Institutes of Biomedical Sciences, Fudan University, Shanghai 200032, China. Email: xiangzhou@fudan.edu.cn

Running title: Activation of AKT by RMRP

Supplementary Figure Legends

Figure S1. RMRP is associated with poor survival of cancer patients. (A) RMRP is expressed at higher levels in tumors compared to normal tissues from the UALCAN database. (B) The Kaplan-Meier analysis shows that high expression of RMRP is correlated with unfavorable overall survival in multiple human cancers.

Figure S2. RMRP boosts growth and migration of wt p53-harboring breast cancer cells. (A) Overexpression of RMRP prompts, while knockdown of RMRP suppresses, MCF-7 cell growth. (B) Overexpression of RMRP prompts, while knockdown of RMRP suppresses, MCF-7 cell migration. (C) Overexpression of RMRP prompts, while knockdown of RMRP suppresses, CAL-51 cell growth. (D) Overexpression of RMRP prompts, while knockdown of RMRP suppresses, CAL-51 cell migration.

Figure S1

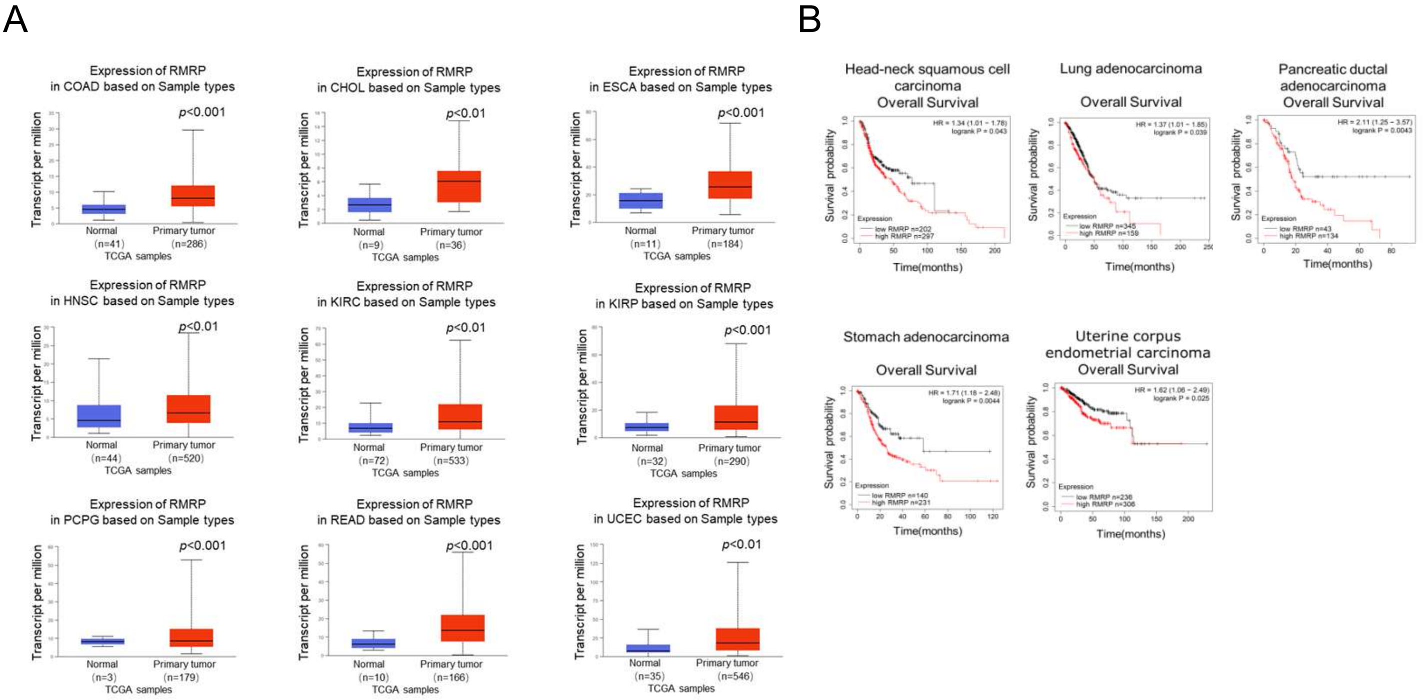
A
Expression of RMRP in COAD based on Sample types
p<0.001
Transcript per million
Normal (n=41)
Primary tumor (n=286)
TCGA samples
Expression of RMRP in CHOL based on Sample types
p<0.01
Transcript per million
Normal (n=9)
Primary tumor (n=36)
TCGA samples
Expression of RMRP in ESCA based on Sample types
p<0.001
Transcript per million
Normal (n=11)
Primary tumor (n=184)
TCGA samples
Expression of RMRP in HNSC based on Sample types
p<0.01
Transcript per million
Normal (n=44)
Primary tumor (n=520)
TCGA samples
Expression of RMRP in KIRC based on Sample types
p<0.01
Transcript per million
Normal (n=72)
Primary tumor (n=533)
TCGA samples
Expression of RMRP in KIRP based on Sample types
p<0.001
Transcript per million
Normal (n=32)
Primary tumor (n=290)
TCGA samples
Expression of RMRP in PCPG based on Sample types
p<0.001
Transcript per million
Normal (n=3)
Primary tumor (n=179)
TCGA samples
Expression of RMRP in READ based on Sample types
p<0.001
Transcript per million
Normal (n=10)
Primary tumor (n=166)
TCGA samples
Expression of RMRP in UCEC based on Sample types
p<0.01
Transcript per million
Normal (n=35)
Primary tumor (n=546)
TCGA samples
B
Head-neck squamous cell carcinoma Overall Survival
Survival probability
Time(months)
Lung adenocarcinoma Overall Survival
Survival probability
Time(months)
Pancreatic ductal adenocarcinoma Overall Survival
Survival probability
Time(months)
Stomach adenocarcinoma Overall Survival
Survival probability
Time(months)
Uterine corpus endometrial carcinoma Overall Survival
Survival probability
Time(months)

Figure S2

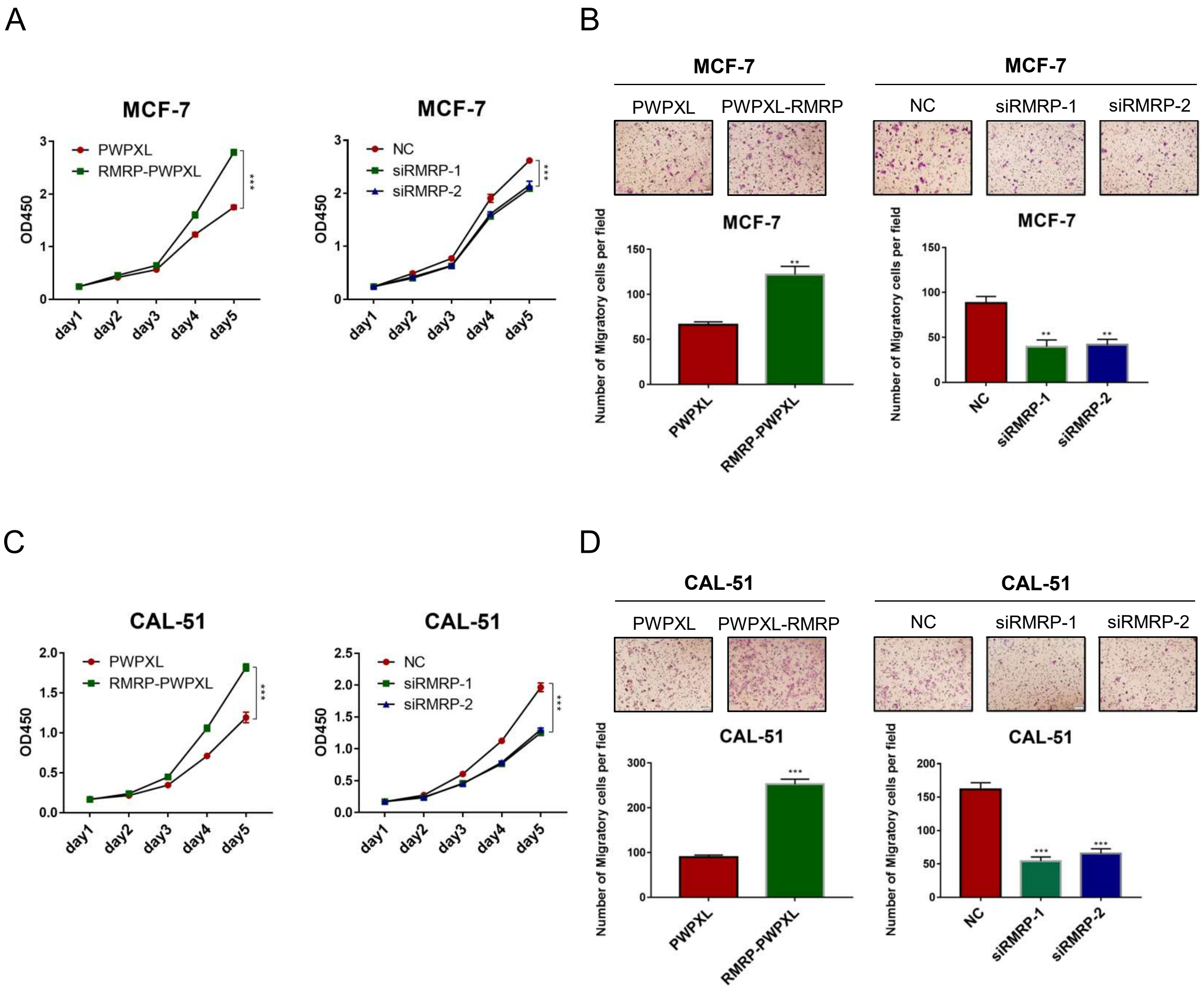
A
MCF-7
PWPXL
RMRP-PWPXL
OD450
day1
day2
day3
day4
day5
***
MCF-7
NC
siRMRP-1
siRMRP-2
OD450
***
B
MCF-7
PWPXL
PWPXL-RMRP
MCF-7
NC
siRMRP-1
siRMRP-2
MCF-7
Number of Migratory cells per field
PWPXL
RMRP-PWPXL
**
MCF-7
Number of Migratory cells per field
NC
siRMRP-1
siRMRP-2
**
C
CAL-51
PWPXL
RMRP-PWPXL
OD450
***
CAL-51
NC
siRMRP-1
siRMRP-2
OD450
***
D
CAL-51
PWPXL
PWPXL-RMRP
CAL-51
NC
siRMRP-1
siRMRP-2
CAL-51
Number of Migratory cells per field
PWPXL
RMRP-PWPXL
***
CAL-51
Number of Migratory cells per field
NC
siRMRP-1
siRMRP-2
***
